# Administration of Ketamine Causes Autophagy and Apoptosis in the Rat Fetal Hippocampus and in PC12 Cells

**DOI:** 10.3389/fncel.2018.00021

**Published:** 2018-02-02

**Authors:** Xinran Li, Yanan Li, Jinghua Zhao, Lina Li, Yuxin Wang, Yiming Zhang, Yue Li, Yu Chen, Wenhan Liu, Li Gao

**Affiliations:** ^1^College of Veterinary Medicine, Northeast Agricultural University, Harbin, China; ^2^Key Laboratory of the Provincial Education, Department of Heilongjiang for Common Animal Disease Prevention and Treatment, Northeast Agricultural University, Harbin, China; ^3^Heilongjiang Key Laboratory for Laboratory Animals and Comparative Medicine, Northeast Agricultural University, Harbin, China

**Keywords:** autophagy, apoptosis, fetus, pregnancy, PC12

## Abstract

Drug abuse during pregnancy is a serious problem. Like alcohol, anticonvulsants, sedatives, and anesthetics, such as ketamine, can pass through the placental barrier and affect the growing fetus. However, the mechanism by which ketamine causes damage to fetal rats is not well understood. Therefore, in this study, we anesthetized pregnant rats with ketamine and evaluated the Total Antioxidant Capacity (T-AOC), Reactive Oxygen Species (ROS), and Malondialdehyde (MDA). Moreover, we determined changes in the levels of Cleaved-Caspase-3 (C-Caspase-3), Beclin-1, B-cell lymphoma-2 (Bcl-2), Bcl-2 Associated X Protein (Bax), Autophagy-related gene 4 (Atg4), Atg5, p62 (SQSTM1), and marker of autophagy Light Chain 3 (LC3). In addition, we cultured PC12 cells *in vitro* to determine the relationship between ROS, autophagy, and apoptosis following ketamine treatment. The results showed that ketamine induced changes in autophagy- and apoptosis-related proteins, reduced T-AOC, and generated excessive levels of ROS and MDA. *In vitro* experiments showed similar results, indicating that apoptosis levels can be inhibited by 3-MA. We also found that autophagy and apoptosis can be inhibited by N-acetyl-L-cysteine (Nac). Thus, anesthesia with ketamine in pregnant rats may increase the rate of autophagy and apoptosis in the fetal hippocampus and the mechanism may be through inhibition of antioxidant activity and ROS accumulation.

## Introduction

Drug abuse during pregnancy is a serious problem (Rofael et al., 2003). Between 0.75 and 2% of pregnant women require surgery that is related to either pregnancy or other medical issues (Goodman, 2007). However, obstetric surgery is complicated and requires increased anesthesia times. Commonly used general anesthetics include N-methyl-D-aspartate (NMDA)-type glutamate receptor blockades or have gamma-aminobutyric acid (GABA) receptor-enhancing properties. Ketamine is a noncompetitive NMDA ion channel blocker (Liu et al., 2013). Preclinical data have suggested that commonly used anesthetics, including ketamine, show neurotoxicity in rodents or in developing brains of primates (Slikker et al., 2007; Satomoto et al., 2009; Brambrink et al., 2012). The developing brains of Rhesus macaques are very sensitive to ketamine, and long-term exposure induced neuronal apoptosis at any age (Brambrink et al., 2012). Ketamine-induced apoptosis of neurons and neurotoxicity has been linked to mitochondria (Bai et al., 2013). However, the mechanism by which ketamine causes toxicity in fetal rats is not well understood.

Recent reports have shown that high doses of ketamine can induce the generation of Reactive Oxygen Species (ROS) in neurons (Ito et al., 2015). ROS have been reported to play an important role in a variety of cellular programs (D'Autréaux and Toledano, 2007). By acting as cellular signaling factors, ROS may trigger the formation and degradation of autophagosomes (Chen et al., 2009). On the contrary, by removing aggregated proteins and damaged organelles (such as mitochondria), autophagy can reduce oxidative damage and ROS levels (Ureshino et al., 2014). In the autophagosome formation process, ROS inhibited the activity of autophagy-related gene 4 (Atg4) control autophagy, and induced Atg4 inactivation, thereby causing Light Chain 3-II (LC3-II) accumulation, leading to increased autophagy (Kaminskyy and Zhivotovsky, 2014; Yadav and Chandra, 2014). Previous studies have shown that oxidative stress-induced protein and organelle damage can induce Beclin1 from the anti-apoptotic protein B-cell lymphoma 2 (Bcl-2), to form a Beclin1-VPS34-Atg14 complex, leading to membrane separation and autophagy nucleation, thereby starting autophagy damaged parts (Valente et al., 2014). Zhao et al. hypothesized that COX5B interacts with ATG5 and inhibits ROS production (Zhao et al., 2012). Due to its interaction with the autophagy marker LC3, p62, which is also known as sequestrome1 (SQSTM1), is one of the most well-studied autophagic receptors (Bitto et al., 2014). A survival mechanism of the switch from endoplasmic reticulum (ER) stress-induced apoptosis to autophagy was initiated via ROS-mediated JNK/p62 signals in methotrexate-resistant choriocarcinoma cells (Shen et al., 2015). However, whether ketamine causes oxidative stress in fetal rats, and the relationship between ROS accumulation, autophagy, and apoptosis is unclear.

Therefore, in this study we anesthetized pregnant rats with ketamine and investigated the effects on Total Antioxidant Capacity (T-AOC), levels of ROS and Malondialdehyde (MDA), and the expression of Cleaved-Caspase-3 (C-Caspase-3), Beclin-1, Bcl-2, Bcl-2 Associated X Protein (Bax), Atg4, Atg5, p62 (SQSTM1), and LC3. Furthermore, PC13 cells were treated with ketamine to evaluate the relationship between ROS, autophagy, and apoptosis.

## Materials and methods

### Animals

Three-month-old male and female Wistar rats weighing 220 ± 20 g were purchased from the Animal Experimental Center of the Second Affiliated Hospital of Harbin Medical University (Harbin, China). Prior to experiments, rats were quarantined for 2 weeks at the Northeast Agricultural University (Harbin, China), and housed in polypropylene cages in a temperature and humidity-controlled chamber with a 12 h light/dark cycle. Rats had access to water and food *ad libitum*. All experiments were performed in accordance with the Ethical Committee for Animal Experiments (Northeast Agricultural University, Harbin, China).

### Mating, drug administration, and sample collection

**Figure 1:**
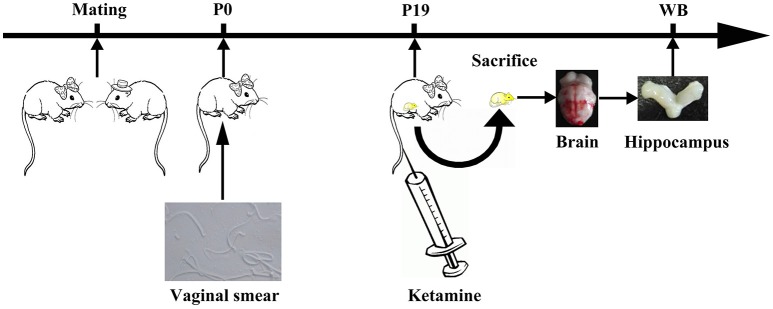
Experimental flow diagram. One male and two female rats were mated in a cage. On The next morning, vaginal suppository was observed under the iron mesh. When sperm was observed by vaginal smear, female rats were annotated as pregnant at day 0 (P0). On P19, female rats were anesthetized by intravenous injection of ketamine (200 mg/kg) for 3 h.

Twenty four female rats and 12 male rats were randomly assigned to 12 cages. If on the following day, before noon, sperm was observed in vaginal smears were observed, if observed sperm, the female rats recorded as pregnant 0 days (P0). Twelve female rats were anesthetized by intravenous injection of ketamine (200 mg/kg) for 3 h on P19. The total volume of ketamine was no more than 2 mL/100 mg (K group, Figure 1). Other twelve female rats (C group) was injected with the same volume of saline. Rats were sacrificed after drug administration and the hippocampus was collected from fetal rats.

### Immunohistochemistry

Hippocampal sections were immersed in a covered plastic container with the target retrieval solution and placed in an autoclave for 15 min at 121°C. Endogenous peroxidase was blocked using a peroxidase blocking reagent for 10 min at room temperature. Rat monoclonal antibody directed against C-Caspase-3 (Abcam Shanghai Trading Co, Ltd., Shanghai, China) diluted in PBS (1:100) was used as the primary antibody. Sections were incubated for 12 h at 4°C. Then, sections were washed with PBS and incubated with a goat anti-rabbit secondary antibody (Abcam Shanghai Trading Co, Ltd., Shanghai, China) was applied for 40 min at room temperature. Diaminobezidine tetra- hydrochloride (Wuxianfeng technology Co, Ltd., Harbin, China) was used as a chromogen, and sections were counterstained with hematoxylin. From each section, three fields were randomly chosen at 100 × magnification, and integrated optical density (IOD, ImagePro Plus 6.0) was used as an indicator of positive substance content.

### Cell culture and drug treatments

The PC12 cell line was derived from pheochromocytoma of the rat adrenal medulla and is widely used as a model system to study neuronal function. Cells were cultured in DMEM (Gibco, Thermo Fisher, Waltham, MA, USA), supplemented with 10% (v/v) FBS (Gibco), penicillin/streptomycin (100 U/mL and 100 μg/mL, respectively) at 37°C in 5% CO_2_. Cells were seeded at a density of 2–9 × 10^5^ cells/well in 6-well plates or at 2–9 × 10^4^ cells/well in 96-well plates andculture medium was changed once daily. Cells were pretreated for 3 h with ketamine (K and H group, 0.6 μg/mL; M group, 0.4 μg/mL; L group, 0.2 μg/mL), H_2_O_2_ (H_2_O_2_ Group, 5 mM), N-Acetyl Cysteine (NAC, 5 mM; Merck, Kenilworth, NJ, USA), Rapamycin (Rapa, 100 nM; Tocris, Bristol, UK), 3-Methyladenine (3-MA, 3 mM; Selleck, Houston, TX, USA) and PBS (C group, similar volume as the corresponding drug).

### Annexin V-FITC/PI staining assay

The annexin V-FITC/PI apoptosis detection kit (BioVision, Milpitas, CA, USA) was used to detect apoptotic cells according to the manufacturer's instructions. PC12 cells (1.5 × 10^5^ cells/well) were seeded into 6-well plates and cultured for 24 h at 37°C. Following incubation, cells were harvested, washed, and re-suspended in 300 μL PBS, containing 3 μL Annexin V-FITC and 3 μL PI for 30 min at room temperature. A total of 10,000 cells were collected and analyzed by flow cytometry. Experiments were performed in triplicate.

### Transfection

The tandem-labeled mRFP-EGFP-LC3 plasmid was purchased from GenePharma (Shanghai, China). Cells were seeded 2–9 × 10^4^ cells/well in 6-well plates and incubated overnight in complete medium. mRFP-EGFP-LC3 was transfected with Lipofectamine® 2000 (Thermo, Shanghai, China) for 6 h at 37°C, replaced with complete medium, and corresponding drugs were added.

### Cell counting kit-8 assay

Cell viability was determined using the CCK-8 assay (Nanjing Jiancheng Bioengineering Institute, Nanjing, China). The optical density of each well was measured at 450 nm. Experiments were performed in triplicate.

### Detection of ROS activity

ROS activity was measured post-transfection using a ROS assay kit (Nanjing Jiancheng Bioengineering Institute, Nanjing, China). Briefly, 10 μM 2,7-dichlorofuresciin (DCFH-DA) was added and samples were incubated at 37°C. Media was discarded and samples were washed three times with wash solution. Cell suspension was prepared by harvesting cells with trypsin. Cell suspension was centrifuged at 1,000 g for 10 min, after which cells were collected and washed 2 times with PBS. ROS activity was measured at 500 nm (excitation wave length) and 530 nm. One sample was taken from every four dams, and measurements were performed in triplicate.

### Determination of antioxidant ability

The protein content ability of T-AOC and the content of MDA were measured using the protein quantitative detection kit (Nanjing Jiancheng Bioengineering Institute, P.R. China) according to the manufacturer's guidelines. One sample was taken from every four dams, and measurements were performed in triplicate.

Antioxidant ability was calculated as follows:

$$\begin{array}{ll} \begin{array}{c} \text{Ability of T-AOC}\\ \left(\text{U}/\text{mgprot}\right) \end{array} & =\frac{\text{Sample OD}-\text{Control OD}}{0.01\times 30}\times \frac{\text{Total volume of liquid}}{\text{Volumeofsampling }}\times \text{Dilution factor}÷\begin{array}{c} \text{Concentration of sampling}\\ \left(\text{mgprot}/\text{ml}\right) \end{array} \end{array}$$

$$\begin{array}{ll} \begin{array}{c} \text{Content of MDA}\\ \left(\text{mmol}/\text{mgprot}\right) \end{array} & =\frac{\text{Sample OD}-\text{Control OD}}{\text{Standard OD }-\text{ Blank OD}}\times \text{Standard concentration}\left(10\text{nmol}/\text{ml}\right)÷\begin{array}{c} \text{Sample concentration}\\ \left(\text{mgprot}/\text{ml}\right) \end{array} \end{array}$$

### Western blot analysis

One sample was taken from every four dams, and measurements were performed in triplicate. Proteins were separated by 10% SDS-polyacrylamide gel electrophoresis and transferred to nitrocellulose membranes (HybondTM-C Extra, GE Healthcare, Little Chalfont, UK). Primary antibodies directed against LC3 (1.5:1,000; EnoGene, Nanjing, China), Cleaved-caspase 3 (1.5:1,000; EnoGene, Nanjing, China), Beclin-1 (1.5:1,000; EnoGene, Nanjing, China), Atg5 (1.5:1,000; EnoGene, Nanjing, China), Bax (1.5:1,000; EnoGene, Nanjing, China), Bcl-2 (1.5:1,000; EnoGene, Nanjing, China), Atg4 (1:1,000, Abcam, Cambridge, UK), or p62 (1:1,000; CST, Shanghai, China) were used in this study. Secondary antibodies, including goat anti-rabbit IgG and goat anti-mouse IgG (1:10,000; EnoGene) were incubated for 45 min at room temperature. The ECL (Pico) detection kit (Thermo, Shanghai, China) was used for signal detection. Densitometry was performed using Adobe Photoshop software. The expression of LC3, C-Caspase-3, Beclin-1, Atg5, Bax, Bcl-2, Atg4, and p62 was determined by calculating the grayscale value ratio to the GAPDH band, and was normalized to the control group.

### Data analysis

Data were analyzed by GraphPad Prism 7.0 (GraphPad Software Inc., La Jolla, CA, USA) and SPSS 20.0 software (SPSS Inc., Chicago, IL, USA) by using one-way ANOVA followed by Tukey's *Post-Hoc* test or unpaired two-tailed Student's *t*-test. Values were considered statistically significant when *P* < 0.05. Data are presented as the mean ± SD unless stated otherwise.

## Results

### The effect of ketamine on the hippocampus

**Figure 2:**
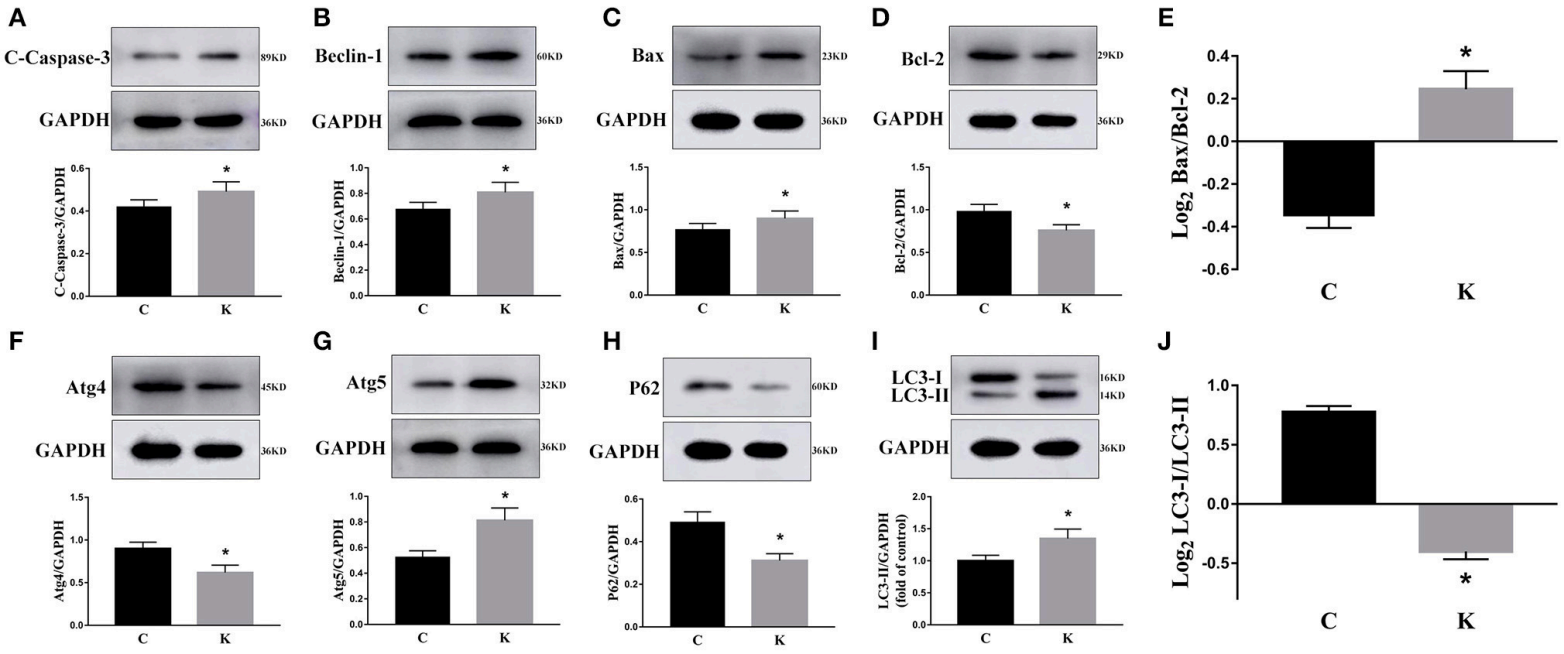
Effects of ketamine on autophagy and apoptosis of the hippocampus in fetal rats. Increase in protein levels of C-Caspase-3 (16.4%) **(A)**, Beclin-1 (18.9%) **(B)**, Bax (16.1%) **(C)**, Atg5 (53.4%) **(G)**, and LC3-II (34.7%) **(I)**. Decrease in protein levels of Bcl-2 (23.2%) **(D)**, Atg4 (20.1%) **(F)**, p62 (36.9%) **(H)**, and LC3-I (40.3%) **(I)** The Bax/Bcl-2 ratio increased **(E)**, whereas the LC3-I/LC3-II ratio decreased **(J)**. ^*^p < 0.05.

**Figure 3:**
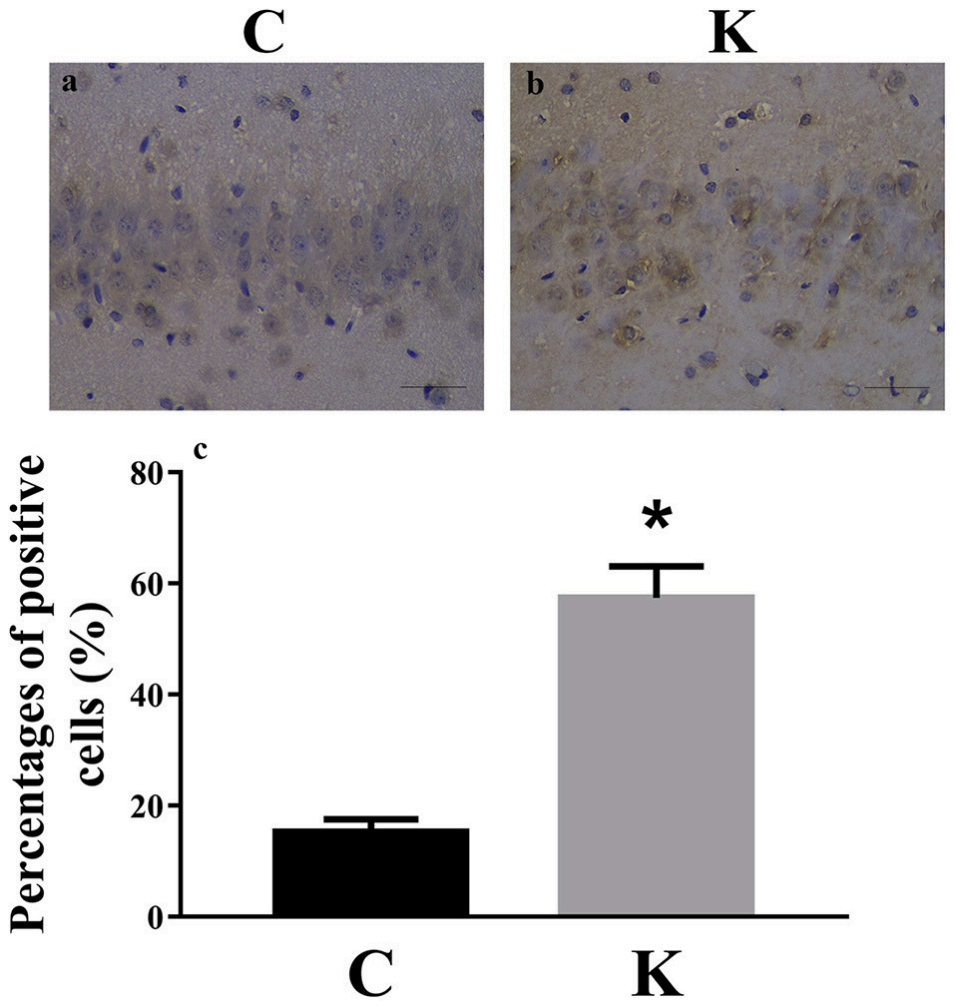
Immunohistochemistry staining of hippocampal CA1 cells in C and K groups to visualize the expression and localization of C-Caspase-3. The expression levels of hippocampal C-Caspase-3 in K group **(a)** significantly increased comparing with C group **(b)**. **(c)** The percentages of C-Caspase-3 positive cells of C group and K group in the hippocampus. Scale-bars: 50 μm. ^*^p < 0.05.

**Figure 4:**
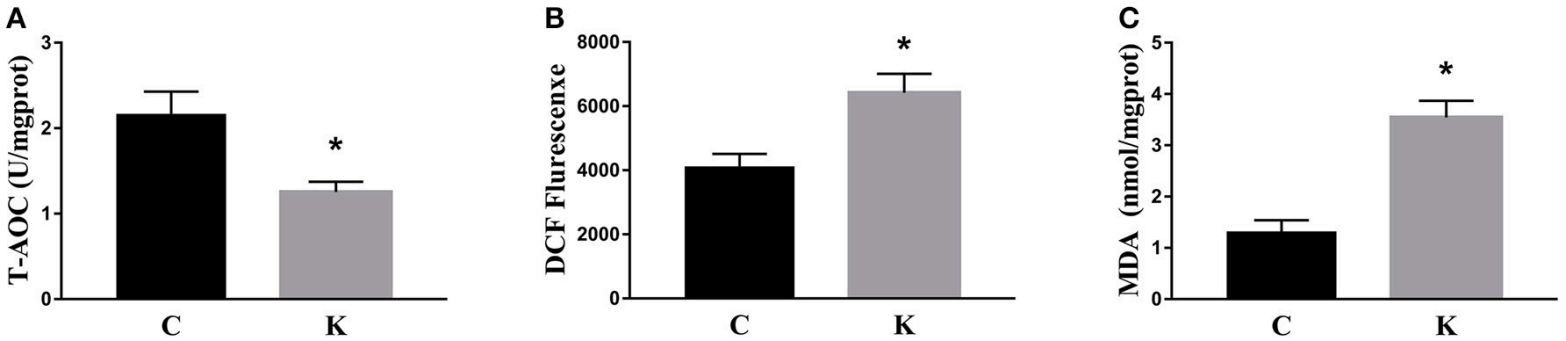
Effects of ketamine on oxidative stress in the hippocampus of fetal rats. Ketamine decreased the ability of T-AOC **(A)**, whereas levels of ROS **(B)**, and MDA **(C)** were increased compared with the C group. ^*^p < 0.05.

We investigated the hippocampal expression levels of proteins involved in apoptosis and autophagy to determine the effect of ketamine on injury. In ketamine-treated rats, increased protein levels were observed for C-Caspase-3 (16.4%), Beclin-1 (18.9%), Bax, (16.1%), Atg5 (53.4%), and LC3-II (34.7%). On the contrary, when compared with the C group, decreases were found in protein levels of Bcl-2 (23.2%), Atg4 (20.1%), p62 (36.9%), and LC3-I (40.3%). Additionally, the Bax/Bcl-2 ratio increased, whereas the LC3-I/LC3-II ratio decreased (Figure 2). The expression levels of hippocampal C-Caspase-3 in K group significantly increased comparing with C group. The percentages of C-Caspase-3 positive cells of C group and K group in the hippocampus (Figure 3). Moreover, the ability of T-AOC decreased while MDA and ROS increased when compared with the C group (Figure 4).

### Effects of ketamine on PC12 cells

**Figure 5:**
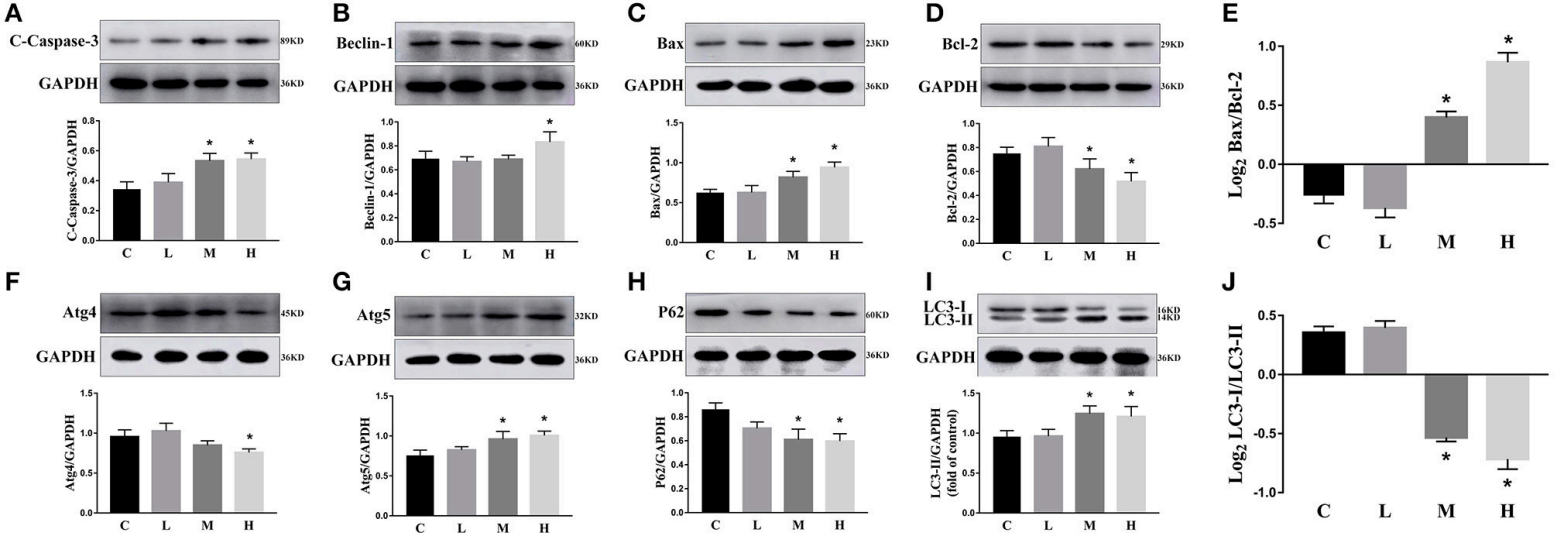
Effects of ketamine on PC12 cells. The protein levels of C-Caspase-3 **(A)**, Beclin-1 **(B)**, Bax **(C)**, Atg5 **(G)**, and LC3-II **(I)** increased after ketamine treatment, whereas Bcl-2 **(D)**, Atg4 **(F)**, p62 **(H)**, and LC3-I **(I)** decreased. The Bax/Bcl-2 ratio increased **(E)** and the LC3-I/LC3-II ratio decreased **(J)** after ketamine treatment. ^*^p < 0.05.

**Figure 6:**
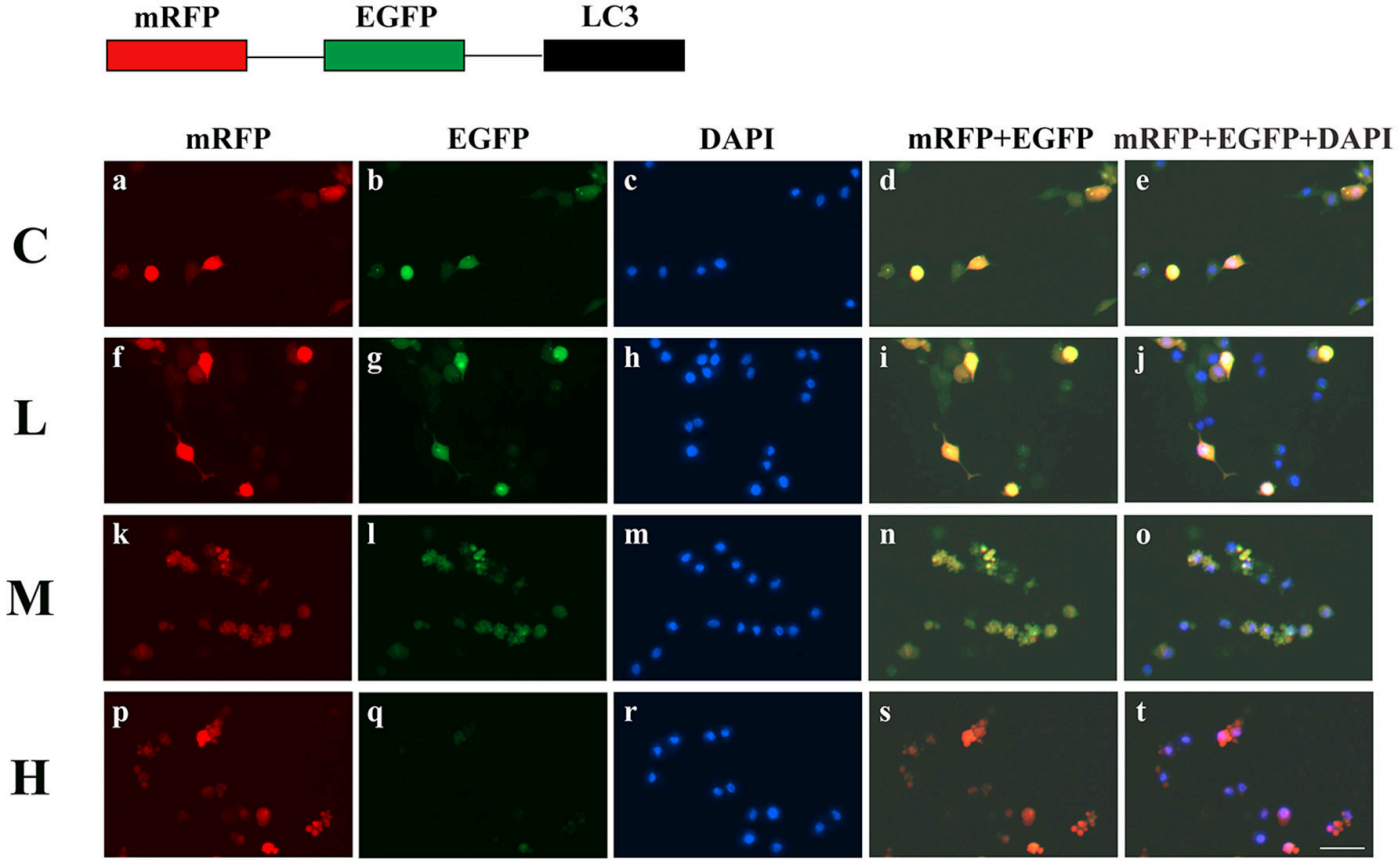
Ketamine-induced autophagy is dose dependent. C **(a–e)**, L **(f–j)**, and M **(k–o)** groups showed a yellow color, which indicated that the eGFP fluorescence was not quenched. However, the H **(p–t)** group showed a red color, indicating autophagy. Moreover, LC3 localized to both the inside and the outside of forming autophagosomes in the M Group. Scale bar: 10 μm.

To confirm the dosage of ketamine on cells, we designed a concentration gradient and performed CCK-8 tests (Table 1). Cell viability was 49, 64, and 76% respectively at ketamine concentrations of 0.6, 0.4, and 0.2 μg/mL. Compared with the C group, protein levels of C-Caspase-3, Bax, Atg5, and LC3-II increased 55.0, 30.7, 26.8, and 29.6%, whereas in the M group Bcl-2 and p62 decreased 17.3 and 29.3%. In the H group, protein levels of C-Caspase-3, Beclin-1, Bax, Atg5, and LC3-II increased 58.3, 20.1, 50.6, 32.1, and 26.3% whereas Bcl-2, Atg4, and p62 decreased 31.2, 20.7, and 30.9%. Moreover, the Bax/Bcl-2 ratio increased 58.2 and 118.7% in the M and H group compared to the C group, respectively. The LC3-I/LC3-II ratio decreased 45.9 and 52.4% in the M and H group when compared to the C group (Figure 5). To further confirm these findings, cells were transfected with a tandem-labeled mRFP-EGFP-LC3 plasmid (Figure 6). When the process of autophagic flux occurred in a normal fashion, red puncta were observed within the cells because of the easy-to-quench EGFP and the relatively stable RFP in an acidic environment (i.e., autolysosome and lysosome), whereas inhibition of autophagosome-lysosome fusion and/or lysosomal function resulted in the most puncta exhibiting both green and red fluorescence, and thus presented as yellow in merged images (Tang et al., 2017). As shown in Figure 6, the C, L, and M groups showed a yellow color, which indicated that the EGFP fluorescence was not quenched. However, the H group exhibited a red color indicating autophagy. Moreover, LC3 was localized to both the inside and outside of forming autophagosomes in the M Group.

**Table 1:** Results of CCK-8 test.

**Concentration of ketamine (μg/mL)**	**1**	**0.9**	**0.8**	**0.7**	**0.6**	**0.5**	**0.4**	**0.3**	**0.2**
Cell viability(%)	17	34	39	43	49	53	64	75	76

### Ketamine causes autophagy and apoptosis in PC12 cells

**Figure 7:**
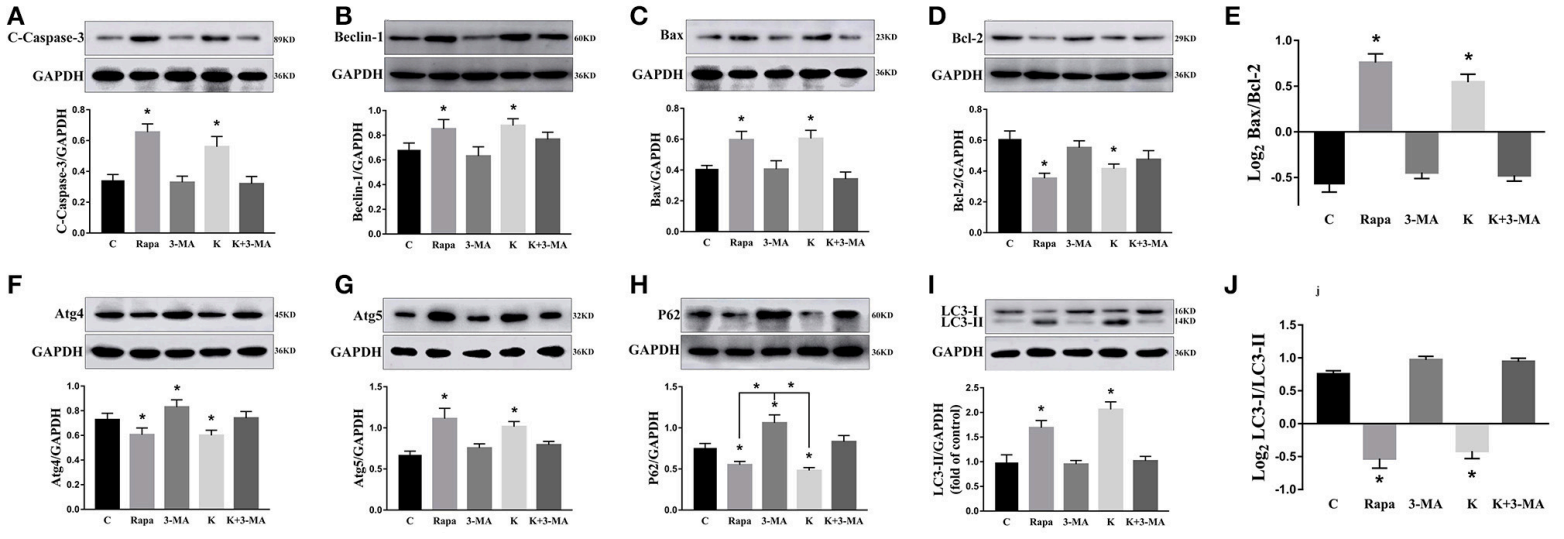
Effects of ketamine on autophagy and apoptosis in PC12 cells. The protein levels of C-Caspase-3 **(A)**, Beclin-1 **(B)**, Bax **(C)**, Atg5 **(G)**, and LC3-II **(I)** increased after Rapa and ketamine treatment, whereas Bcl-2 **(D)**, Atg4 **(F)**, p62 **(H)**, and LC3-I **(I)** decreased. 3-MA resulted in accumulation of p62 **(H)**. In Rapa and K group, the values of log_2_ Bax/Bcl-2 were significantly higher than those in C group **(E)**, while the values of Log_2_LC3-I/LC3-II were significantly lower than those in C group **(J)**. ^*^p < 0.05.

**Figure 8:**
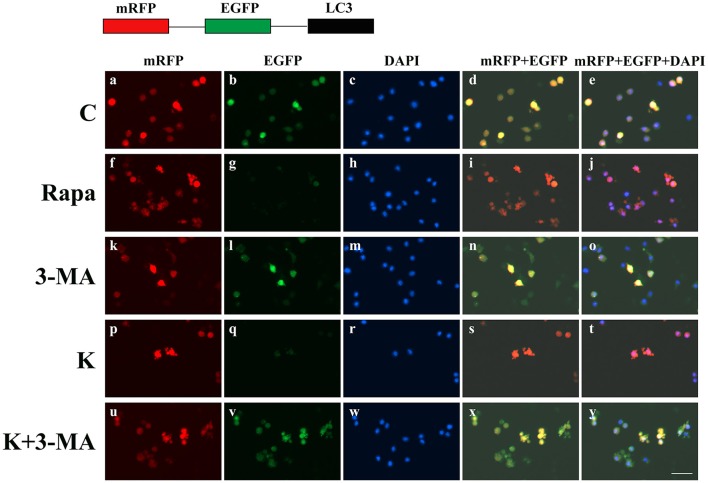
Ketamine-induced autophagy can be blocked by 3-MA. C **(a–e)**, 3-MA **(k–o)**, and K+3-MA **(u–y)** groups showed a yellow color, which indicated that the eGFP fluorescence was not quenched. Ketamine **(p–t)** and Rapa **(f–j)** induced cell autophagy after transfection with the mRFP-EGFP-LC3 plasmid. LC3 localized to autophagosomes, however autophagosomes failed to bind to lysosomes. Scale bar: 10 μm.

To further validate whether ketamine induces cell autophagy directly, we used the autophagic inducer Rapa and inhibitor 3-MA to treat cells. We found that ketamine and Rapa had similar effects on autophagy related genes, while 3-MA treatment led to accumulation of p62 (Figure 7). Compared to the C group, protein levels of C-Caspase-3, Beclin-1, Bax, Atg5, and LC3-II increased 91.2, 24.6, 46.4, 65.7, and 71.6%, respectively, whereas protein levels of Bcl-2, Atg4, and p62 decreased 42.1, 16.8, and 27.2% in the Rapa group. In the 3-MA group, protein levels of Atg4 and p62 increased 14.0 and 40.4%, respectively. Moreover, the protein level of C-Caspase-3, Beclin-1, Bax, Atg5, and LC3-II increased 63.5, 28.7, 48.4, 32.5, and 109.1%, whereas the protein level of Bcl-2, Atg4 and p62 decreased 31.7, 16.7, and 36.0% in the K group. The Bax/Bcl-2 ratio increased 152.3 and 117.5% in the Rapa and K group when compared to the C group, respectively and the LC3-I/LC3-II ratio decreased 58.9 and 55.5% in the Rapa and K group when compared to the C group, respectively. Furthermore, ketamine and Rapa also induced cellular autophagy after transfection with the mRFP-EGFP-LC3 plasmid. Additionally, LC3 was localized to autophagosomes, however, autophagosomes failed to bind to lysosomes (Figure 8).

### Ketamine causes autophagy in PC12 cells by oxidative damage

**Figure 9:**
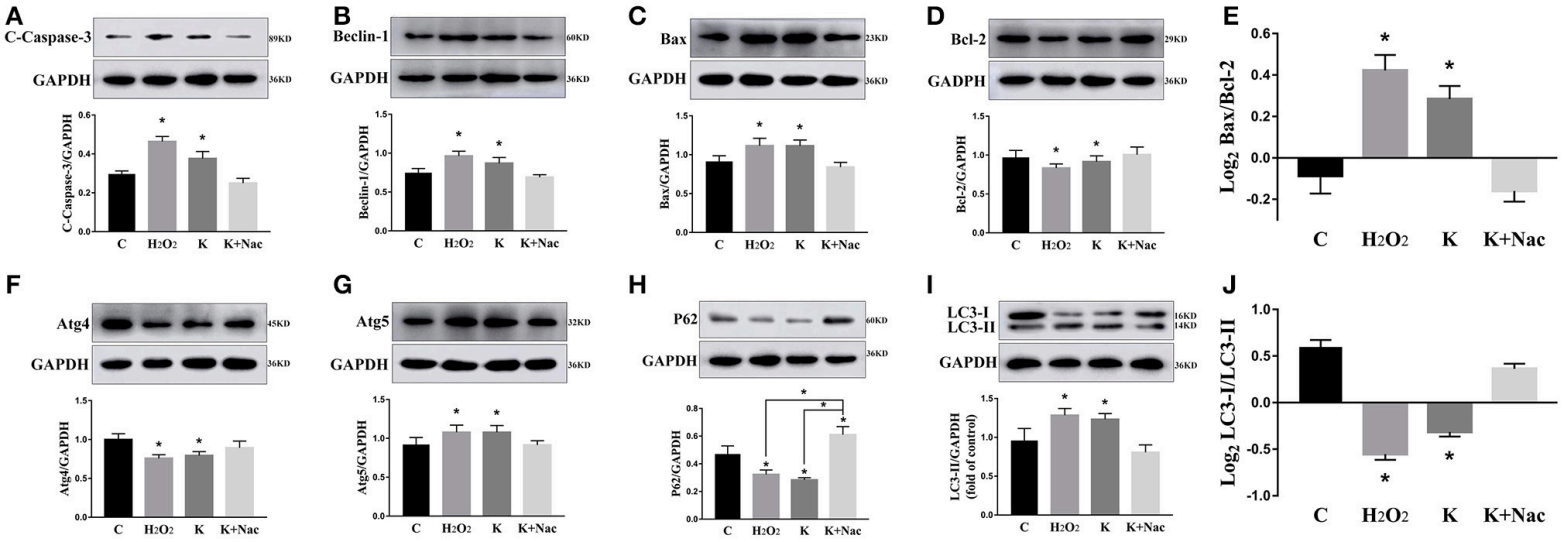
Ketamine induces autophagy in PC12 cells by oxidative damage. Ketamine and H_2_O_2_ induced changes in autophagy-related protein. The protein levels of C-Caspase-3 **(A)**, Beclin-1 **(B)**, Bax **(C)**, Atg5 **(G)**, and LC3-II **(I)** increased after H_2_O_2_ and ketamine treatment, whereas Bcl-2 **(D)**, Atg4 **(F)**, p62 **(H)**, and LC3-I **(I)** decreased. In H_2_O_2_ and K group, the values of log_2_ Bax/Bcl-2 were significantly higher than those in C group **(E)**, while the values of Log_2_LC3-I/LC3-II were significantly lower than those in C group **(J)**. ^*^p < 0.05.

**Figure 10:**
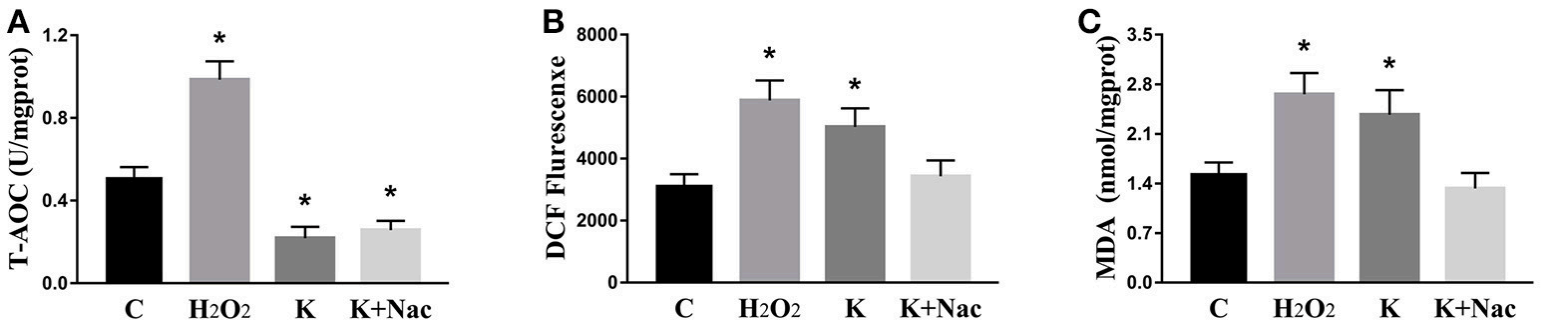
Effects of ketamine on oxidative stress in PC12 cells. In the K group and K+Nac group, T-AOC decreased **(A)**, whereas no significant changes were found in ROS activities **(B)** or MDA content **(C)** in the H_2_O_2_ and K groups. ^*^p < 0.05.

**Figure 11:**
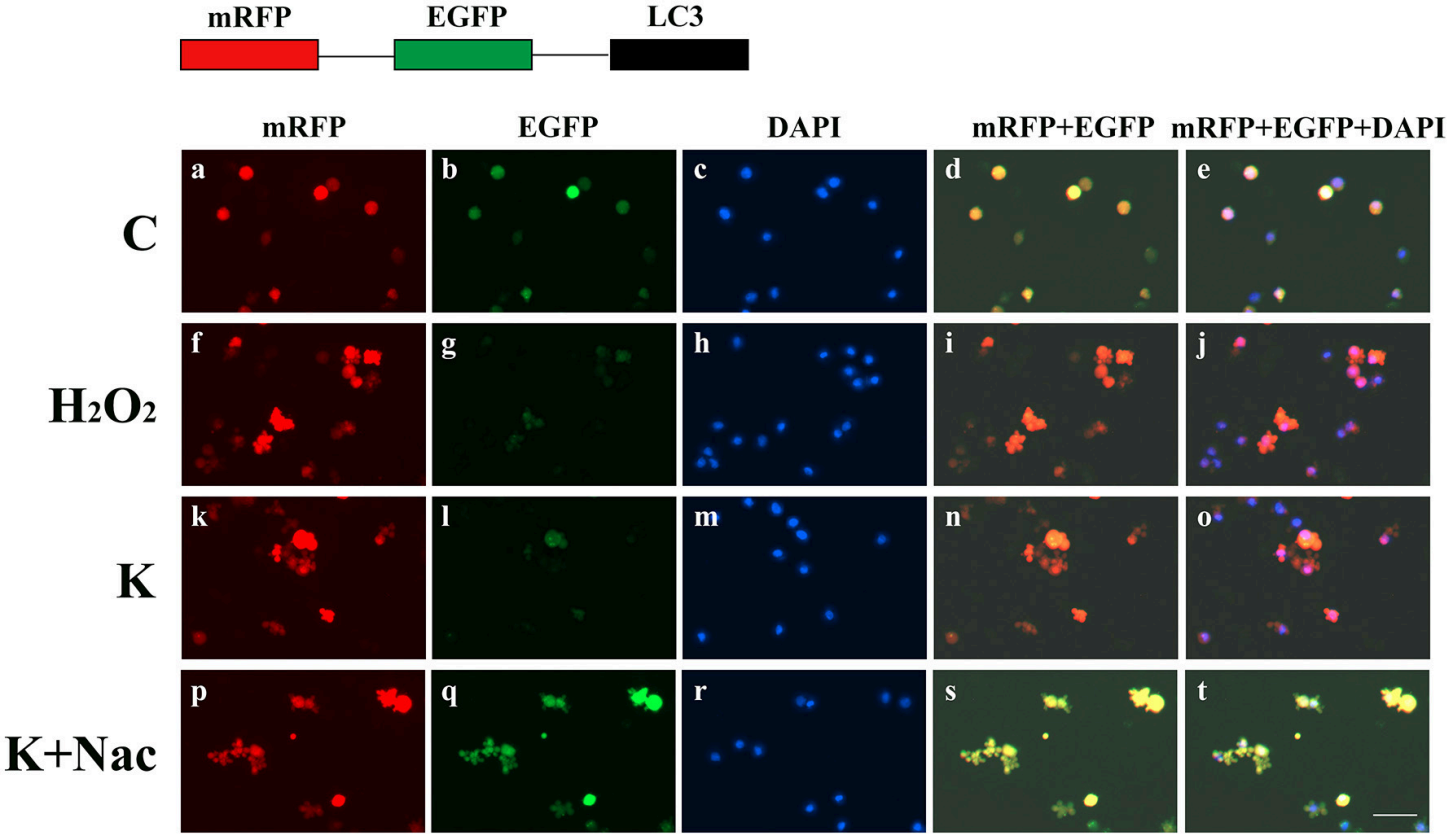
Ketamine-induced autophagy can be blocked by treatment with Nac. C **(a–e)**, and K+Nac **(p–t)** groups showed a yellow color, which indicated that the eGFP fluorescence was not quenched. Ketamine **(k–o)** and H_2_O_2_ **(f–j)** induced apoptosis after transfection with the mRFP-EGFP-LC3 plasmid. LC3 localized to autophagosomes, however autophagosomes failed to bind to lysosomes **(t)**. Scale bar: 10 μm.

**Figure 12:**
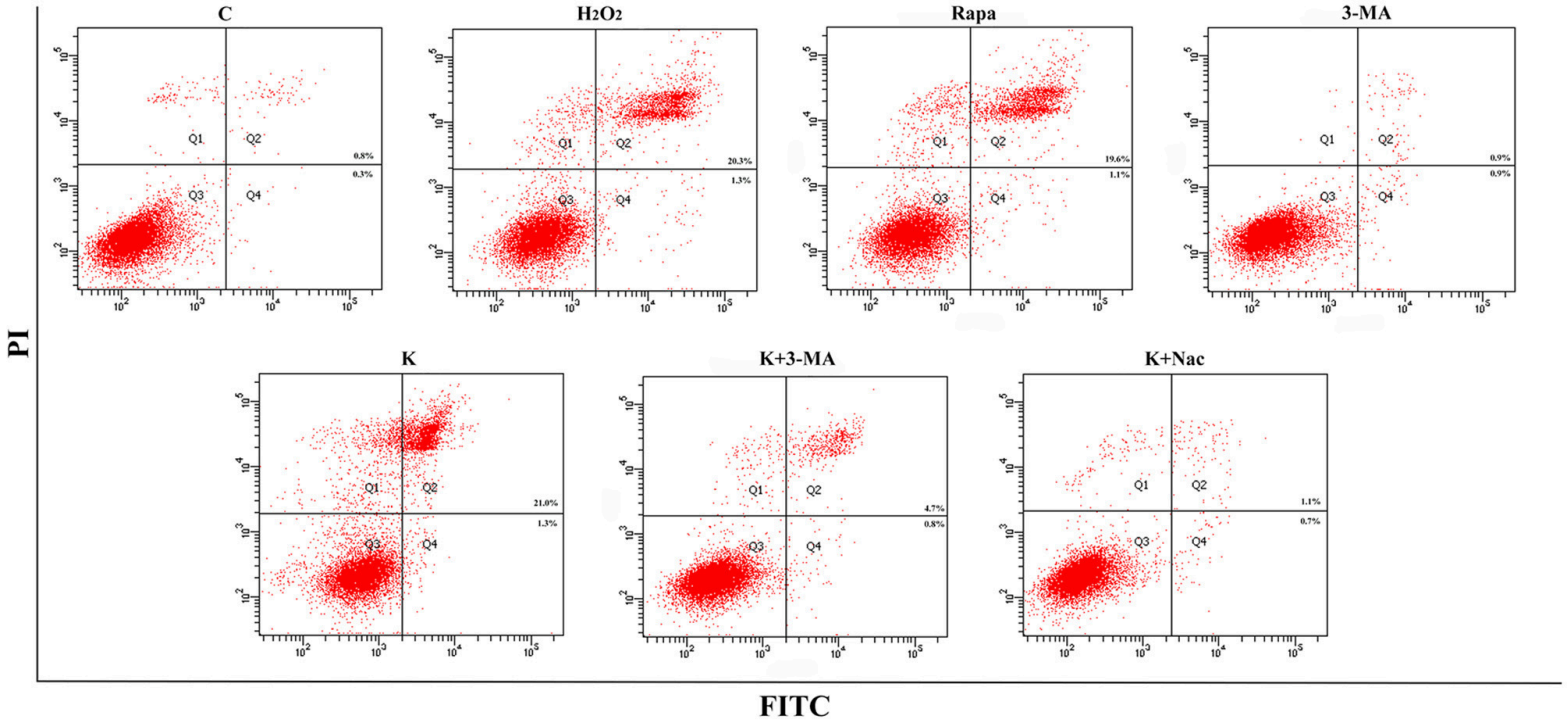
Ketamine induces apoptosis by autophagy. The number of apoptotic cells drastically increased after treatment of cells with H_2_O_2_, Rapa, or Ketamine.

To further explore whether 0.6 μg/mL ketamine induced autophagy through oxidative damage, we used H_2_O_2_ as a positive control. Compared with the C group, we found that both ketamine and H_2_O_2_ induced changes in the levels of autophagy-related proteins (Figure 9). Compared to the C group, the protein levels of C-Caspase-3, Beclin-1, Bax, Atg5, and LC3-II increased 55.9, 28.6, 21.9, 16.3, and 33.5%, whereas the protein levels of Bcl-2, Atg4, and p62 decreased 14.4, 24.0, and 31.3% in the H_2_O_2_ group. Protein levels of C-Caspase-3, Beclin-1, Bax, Atg5, and LC3-II increased 26.6, 16.2, 21.9, 16.3, and 29.5%, whereas protein levels of Bcl-2, Atg4, and p62 decreased 5.9, 20.2, and 40.4% in the K group. The p62 protein level increased 29.8% in the K+Nac group. Furthermore, the activity of T-AOC decreased in both the K and K+Nac group, whereas no significant changes were observed in ROS activity and MDA content in the H_2_O_2_ and K groups (Figure 10). We also used flow cytometry analysis to detect apoptosis. As shown in Figure 12, the number of apoptotic cells drastically increased when cells were treated with H_2_O_2_ or ketamine (*P* = 0.0027). Furthermore, ketamine and H_2_O_2_ induced apoptosis after transfection with the mRFP-EGFP-LC3 plasmid. Additionally, LC3 localized to autophagosomes, however autophagosomes failed to bind to lysosomes (Figure 11).

## Discussion

In our previous studies, we showed that learning and memory of spatial and condition were impaired of rat pups, when pregnant rats were anesthetized with ketamine (Li Y. et al., 2017; Li L. et al., 2017). We demonstrated that the use of ketamine in the female rat reduced nerve density and dendritic spines density in the hippocampus of offspring rats, which influenced the nerve conduction efficiency and learning and memory capacity. Therefore, we hypothesized that the effect on fetal rat hippocampus would be more pronounced when ketamine was administered to pregnant rats. Therefore, the fetal rat hippocampus was chosen as the study object. Ketamine induced changes in expression of autophagy- and apoptosis-related proteins, reduced the T-AOC activity, and generated excessive levels of ROS and MDA. *In vitro* experiments revealed similar results and autophagy and apoptosis appeared to be dose-dependent. Rapa and 3-MA were used to confirm the results seen with ketamine treatment in PC12 cells. Interestingly, apoptosis was inhibited by 3-MA. We also found that autophagy and apoptosis were inhibited by Nac. However, the activity of T-AOC was significantly different in the K+Nac group when compared with the C group. We concluded that ketamine negatively affected fetal rats.

The prevalence of substance abuse in pregnant women is similar to the general population, resulting in high fetal exposure rates during the most vulnerable period regarding neurodevelopment and organogenesis. Many studies have shown that alcohol passes through the placental barrier and impacts the baby (Singer et al., 2016). Anticonvulsants, sedatives, or anesthetics such as ketamine also pass through the placental barrier and may harm the fetus (Zhao et al., 2014).

Previous studies have reported that ketamine damages nerve cells (Li X. et al., 2017). Using 1 mg/kg of ketamine in young male monkeys led to permanent and irreversible damage to brain function (Sun et al., 2014). Moreover, administration of ketamine to pregnant rats resulted in cognitive disorders (Li Y. et al., 2017; Li L. et al., 2017). Ketamine also inhibited the proliferation of nerve cells *in vitro* (Wu et al., 2014). In this study, we showed that ketamine has a dose-dependent effect on PC12 cells. Cells form autophagosomes when treated with 0.4 μg/mL ketamine, however the autophagosomes did not bind the lysosome. Moreover, when treated with 0.6 μg/mL ketamine, green fluorescence was quenched in an mRFP-EGFP-LC3 expression system indicating that autophagosomes fused with lysosomes (Figure 6). Autophagy also occurred in skeletal muscle following ketamine treatment (Kashiwagi et al., 2015). Anesthetic doses of ketamine induced apoptosis in rat neurons (Brown et al., 2015). Taken together, the basis of neurotoxicity induced by ketamine may be linked to high levels of ROS generation (Ito et al., 2015).

Autophagy is a major mechanism used to combat oxidative stress (Azad et al., 2009). In the early stages of oxidative stress or during minor oxidative damage, lysosomes exerted a protective effect (Lemasters, 2005). However, when damaged organelles were removed, such as by impaired mitochondria and ER, autophagy restricted the accumulation of ROS (Azad et al., 2009). LC3-I can be cleaved during the process of autophagy and become covalently bound to phosphatidylethanolamine to form LC3-II (Klionsky et al., 2008). LC3 is normally located in the cytoplasm but localizes to both the inside and outside of forming autophagosomes after processing (Mizushima et al., 2001). Thus, LC3 staining can be used as a marker to evaluate levels of autophagy. In this study, green fluorescence was quenched in the K and Rapa groups, as indicated by a red color (Figure 7). Moreover, the levels of autophagy-related proteins Beclin-1, Atg4, Atg5, p62, and LC3 changed after ketamine treatment (Figure 7). Beclin-1 is an important protein in the autophagy process and Atg5 is essential for autophagosome formation. Beclin-1 can regulate the main step in autophagy, including the formation and maturation of autophagosomes (Gu et al., 2017; Li L. et al., 2017). Atg4, a cysteine protease, plays important roles in the process of autophagosome formation by regulating the disulfide bond between Cys338 and Cys394, and is restored by Trx effectively (Sánchez-Wandelmer et al., 2017). This implies that Atg4 plays important roles in the oxidative reduction process. During autophagosome formation, ROS regulates autophagy through inhibiting Atg4, which causes LC3-II accumulation, and thereby increases the number of autophagosomes (Vidoni et al., 2017). Autophagy substrates are targeted for degradation when they are associated with p62, a multidomain protein that interacts with the autophagy machinery (Bitto et al., 2014). In addition, p62 functions as a selective autophagy receptor for degradation of ubiquitinated substrates (Katsuragi et al., 2015). Interestingly, the expression of p62 was significantly increased in the 3-MA treated group when compared with the C group (Figure 7).

Autophagy and apoptosis have many common regulatory factors, such as the Bcl-2 protein family and Caspase-3 (Marino et al., 2014). In this study, levels of both C-Caspase-3 and Bax/Bcl-2 increased when treated with Rapa, but had opposite effects when treated with 3-MA (Figure 7). These data indicated that autophagy may promote apoptosis. During autophagy, ATP levels increased, thereby exposing intracellular phosphatidylserine, which released apoptotic signals (Ito et al., 2005). The Bcl-2 family of proteins play key and dual roles in both apoptosis and autophagy (Liu et al., 2017). Moreover, the combination of Bcl-2 and Bax directly regulated apoptosis and autophagy and Beclin-1 activated autophagy by binding to Bcl-2/Bcl-xL to form Beclin-1:Bcl-2/Bcl-xL compounds (Marino et al., 2014). However, Beclin-1 can be inactivated through cleavage by caspase-3 (Fu et al., 2013).

As a signal molecule, ROS activated autophagy or apoptosis (Suzuki et al., 2015). Autophagy induced the degradation of catalase, leading to accumulation of ROS in the mitochondria, and damage to DNA, proteins, and lipids, resulting in cell death (Fiers et al., 1999). Additionally, ketamine induced the generation of ROS, which allowed for excessive calcium to enter neurons (Liu et al., 2013). In this study, we demonstrated similar results (Figures 4, 10). We found that ketamine inhibited T-AOC, which did not return to normal levels by the action of Nac. In addition, autophagy and apoptosis occurred in PC12 cells treated with H_2_O_2_ (Figures 9, 11). Notably, p62 levels significantly increased when treated with Nac, which may be due to the action of Nac that inhibited p62 participation in autophagy, resulting in p62 accumulation and reducing the impact of ketamine. However, the exact mechanism of action remains unclear. The level of ROS in PC12 may influence apoptosis mediated by the JNK-p53 signaling pathway (Lu et al., 2017). In addition, ROS may participate in the process of mitochondrial fission. Fission inhibitors reduced the generation of ROS induced by high doses of glucose (Yu et al., 2006). Thus, mitochondrial fission may be one of the mechanisms by which ketamine functions, by producing excess ROS leading to apoptosis. However, in the current study, we did not explore how ketamine induces mitochondrial fission through ROS. Extensive experiments will be carried out in the future to validate these findings.

In conclusion, anesthesia using ketamine in pregnant rats increased autophagy and apoptosis rates in the fetal hippocampus, and the mechanism may involve inhibition of antioxidant activity and accumulation of ROS.

## Author contributions

XL and LG designed the study. XL, YaL, YC, and WL processed the brain tissue. XL and YaL collected and analyzed data. XL, YaL, and LG interpreted the data. XL wrote and edited the manuscript. All authors critically reviewed content and approved final version for publication.

### Conflict of interest statement

The authors declare that the research was conducted in the absence of any commercial or financial relationships that could be construed as a potential conflict of interest.
